# Estimation of ENPP1 deficiency genetic prevalence using a comprehensive literature review and population databases

**DOI:** 10.1186/s13023-022-02577-2

**Published:** 2022-12-02

**Authors:** Lauren M. Chunn, Jeffrey Bissonnette, Stefanie V. Heinrich, Stephanie A. Mercurio, Mark J. Kiel, Frank Rutsch, Carlos R. Ferreira

**Affiliations:** 1 Genomenon, Inc., Ann Arbor, MI 48109 USA; 2 Department of General Paediatrics, Muenster University Children’s Hospital, Münster, Germany; 3 Metabolic Medicine Branch, National Human Genome Research Institute, National Institutes of Health, Bethesda, MD USA

**Keywords:** ENPP1 deficiency, Generalized arterial calcification of infancy (GACI), Autosomal recessive hypophosphatemic rickets type 2 (ARHR2), Population database, Prevalence

## Abstract

**Background:**

ENPP1 Deficiency—caused by biallelic variants in *ENPP1—*leads to widespread arterial calcification in early life (Generalized Arterial Calcification of Infancy, GACI) or hypophosphatemic rickets in later life (Autosomal Recessive Hypophosphatemic Rickets type 2, ARHR2). A prior study using the Exome Aggregation Consortium (ExAC)—a database of exomes obtained from approximately 60,000 individuals—estimated the genetic prevalence at approximately 1 in 200,000 pregnancies.

**Methods:**

We estimated the genetic prevalence of ENPP1 Deficiency by evaluating allele frequencies from a population database, assuming Hardy–Weinberg equilibrium. This estimate benefitted from a comprehensive literature review using Mastermind (https://mastermind.genomenon.com/), which uncovered additional variants and supporting evidence, a larger population database with approximately 140,000 individuals, and improved interpretation of variants as per current clinical guidelines.

**Results:**

We estimate a genetic prevalence of approximately 1 in 64,000 pregnancies, thus more than tripling the prior estimate. In addition, the carrier frequency of *ENPP1* variants was found to be highest in East Asian populations, albeit based on a small sample.

**Conclusion:**

These results indicate that a significant number of patients with ENPP1 Deficiency remain undiagnosed. Efforts to increase disease awareness as well as expand genetic testing, particularly in non-European populations are warranted, especially now that clinical trials for enzyme replacement therapy, which proved successful in animal models, are underway.

**Supplementary Information:**

The online version contains supplementary material available at 10.1186/s13023-022-02577-2.

## Introduction

Ectonucleotide pyrophosphatase/phosphodiesterase 1 (ENPP1; OMIM #173,335) is a transmembrane protein responsible for cleaving ectonucleotides, predominantly adenosine triphosphate (ATP), to generate adenosine monophosphate (AMP) and pyrophosphate (PPi). ENPP1 represents the main contributor to systemic and local concentrations of PPi, a potent inhibitor of hydroxyapatite deposition [1, 2]. ENPP1 Deficiency can result in Generalized Arterial Calcification of Infancy (GACI; OMIM #208,000) which is characterized by ectopic mineralization, particularly along the internal elastic lamina of large and medium-sized arteries, as well as periarticular calcification. Additional arterial involvement stems from intimal proliferation, with consequent luminal narrowing. The constellation of vascular findings leads to organ ischemia, with cardiac failure a common presentation of the disease. Mortality is high, with approximately half of all infants dying from the disease within the first few months of life [2]. In addition, ENPP1 Deficiency can result in Autosomal Recessive Hypophosphatemic Rickets Type 2 (ARHR2; OMIM #613,312) in those who survive GACI, or in individuals who never had clinical cardiovascular manifestations. This form of hypophosphatemic rickets is mediated by FGF23 (OMIM #605,380), a hormone that increases renal losses of phosphate, but the etiology of FGF23 increase remains unknown as of yet [3]. Finally, adult patients present with musculoskeletal symptoms, representing a major cause of morbidity in later life [4].

ENPP1 Deficiency as a whole is known to be a rare disease—defined as a disease affecting less than 200,000 individuals in the United States—but arriving at an accurate estimate of the prevalence is challenging. It is crucial that these estimates are as accurate as possible in order to appropriately assess disease burden as well as the number of patients who may benefit from newly developed therapies. Traditional methods of estimating prevalence rely on clinical data, the accuracy of which can be negatively impacted by diagnostic difficulties, especially for rare diseases with nonspecific and/or heterogeneous phenotypes. For ENPP1 Deficiency, clinical heterogeneity, low clinical awareness, and a high infant mortality rate all contribute to the difficulty associated with estimating the disease prevalence, as clinical data can be unreliable and result in an estimate that is insufficiently representative of the actual patient population.

One method to overcome these diagnostic challenges in estimating prevalence would be to base the estimate on genetic, rather than clinical, data. One such technique based on genetic data involves evaluating the frequency of pathogenic variants in population databases, and then estimating the genetic prevalence of the disease assuming Hardy–Weinberg equilibrium [5–7]. The accuracy of this technique relies on the completeness of the knowledgebase of causative variants as well as the number of healthy individuals contained within the population database that is utilized. A prior study using this technique estimated the genetic prevalence of ENPP1 Deficiency at approximately 1 in 200,000 [3].

In this study, we improve on the previous estimate by performing a more comprehensive literature review using Mastermind to identify additional variants as well as supporting evidence, by using clinical standard variant interpretation to assess the level of evidence supporting the inclusion of variants in the estimate, and by using a larger population database [8].

## Results

To begin determining ENPP1 Deficiency prevalence estimates, *ENPP1* variants were identified through a comprehensive literature review performed using the Mastermind Genomic Search Engine [8]. A total of 183 *ENPP1* variants were identified and interpreted according to the American College of Medical Genetics and Association of Molecular Pathologists (ACMG/AMP) variant interpretation guidelines [9]. Of these variants, 77 were classified as Pathogenic/Likely Pathogenic, 91 as Variants of Undetermined Significance (VUS), 13 as Benign/Likely Benign and 2 as conflicting (having sufficient evidence to meet both a Benign and Pathogenic classification). All Benign/Likely Benign and conflicting variants were excluded from the proceeding analysis.

**Fig. 1:**
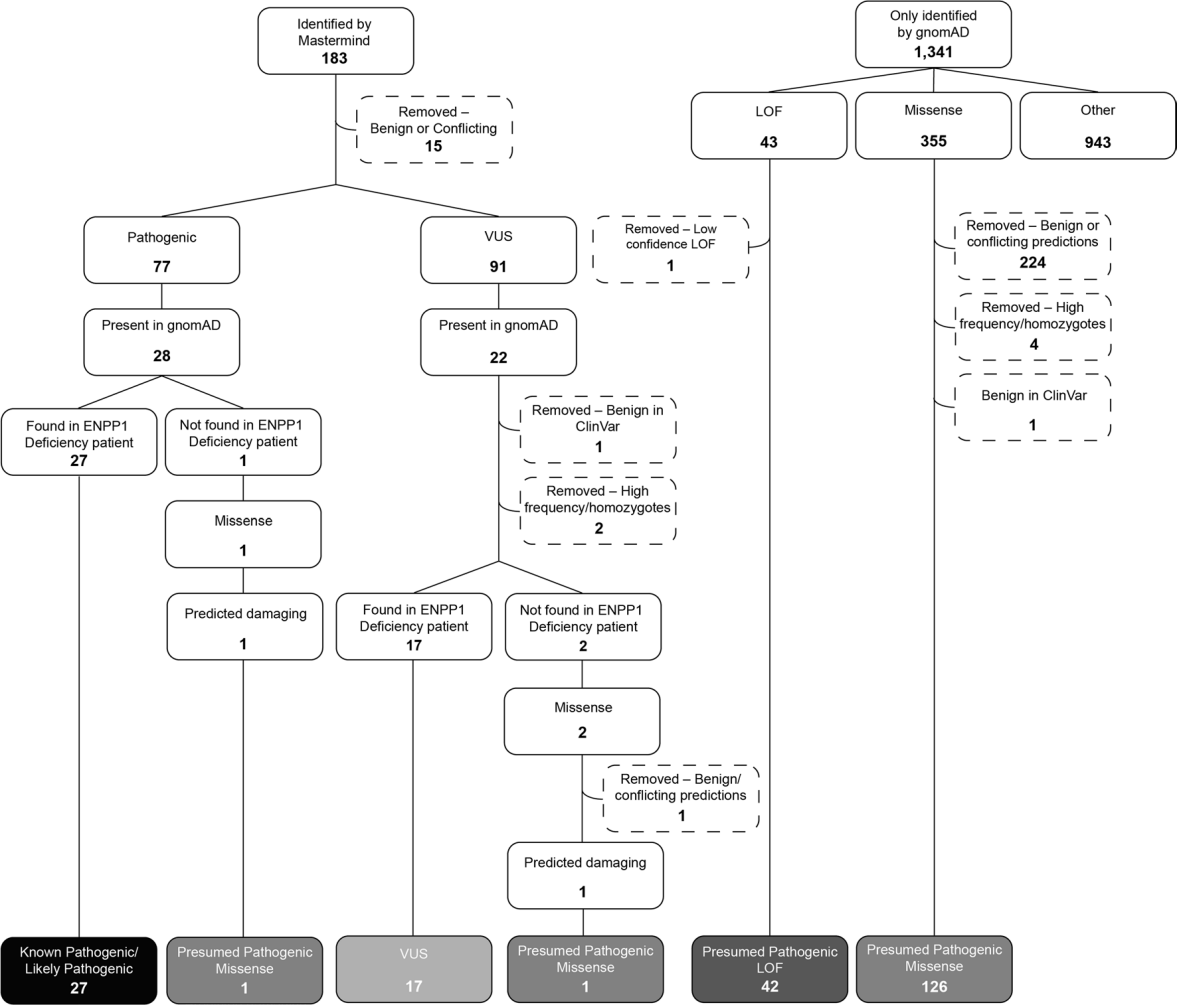
Inclusion/Exclusion of Variants in *ENPP1.* The number of variants included or excluded, along with the reason, is displayed. Loss of function (LOF) variants include start loss, nonsense, frameshift, and canonical splice site variants. *VUS* Variant of Undetermined Significance

Variants classified as Pathogenic/Likely Pathogenic or VUS that had a non-zero allele frequency in the Genome Aggregation Database (gnomAD) were included or excluded as described in Additional file 1: Figs. S1 and S2, respectively [10]. This selection process considered the variant’s presence in ENPP1 Deficiency patients, allele frequency and presence in homozygotes in gnomAD, classification in ClinVar, effect type, and predictions from computational algorithms [11]. Following this process, a total of 27 Pathogenic/Likely Pathogenic variants and 17 VUSs were included in the prevalence calculation as Known Pathogenic/Likely Pathogenic variants and VUS variants, respectively (Fig. 1; Additional file 2: Table S1). Notably, 49 of the total Pathogenic/Likely Pathogenic variants (64%) and 69 of the total VUS (76%) were excluded solely as a result of not being present in gnomAD.

In addition, there were variants in the gnomAD database that were not identified in the published literature, which were included or excluded as described in Additional file 1: Fig. S3. This selection process considered the variant’s allele frequency and presence in homozygotes in gnomAD, classification in ClinVar, effect type, and predictions from computational algorithms. Following this process, a total of 42 Presumed Pathogenic Loss of Function (LOF) variants and 128 Presumed Pathogenic missense variants were included in the prevalence calculation (Fig. 1; Additional file 2: Table S1).

The overall allele frequency in gnomAD was summed across the specific groups of included variants—Known Pathogenic/Likely Pathogenic, VUS, Presumed Pathogenic LOF, and Presumed Pathogenic Missense variants. The carrier frequency and genetic prevalence was then calculated using the Hardy–Weinberg equilibrium equation for these groups to showcase the range of estimates that result from different levels of stringency in the variant selection process.

The carrier frequency for *ENPP1* variants associated with ENPP1 Deficiency was estimated to be 1 in 509 to 1 in 127 in the general population which corresponds to a genetic prevalence of 1 in 1,033,927 to 1 in 64,035 pregnancies (Table 1).

**Table 1:** Genetic Prevalence of ENPP1 Deficiency

	Known pathogenic/Likely pathogenic	Known pathogenic/Likely pathogenic and LOF presumed pathogenic	Known pathogenic/Likely pathogenic and all presumed pathogenic
VUS excluded	1/1,033,927	1/334,177	1/82,620
VUS included	1/471,585	1/206,123	1/64,035

The genetic prevalence was calculated using the Hardy–Weinberg equilibrium equation and the sum of the allele frequencies of the specified variants. The ratio represents the proportion of pregnancies that have an ENPP1 Deficiency associated genotype

*Known Pathogenic/Likely Pathogenic* variants that were classified as Pathogenic/Likely Pathogenic by ACMG/AMP

*LOF Presumed Pathogenic* start loss, nonsense, frameshift, and canonical splice site variants that were found in gnomAD but not the published literature

*All Presumed Pathogenic* LOF Presumed Pathogenic variants in addition to predicted damaging missense variants that were found in gnomAD but not the published literature

There was a 1,515% difference between the lowest, most conservative prevalence estimate, which included only variants known to be Pathogenic/Likely Pathogenic by ACMG/AMP, and the highest, most inclusive prevalence estimate, which included all Presumed Pathogenic variants as well as VUS. Notably, the inclusion of Presumed Pathogenic LOF and Presumed Pathogenic Missense variants had a large impact on the estimated prevalence. Inclusion of Presumed Pathogenic LOF variants increased the estimate (excluding VUSs) by 209% which increased by another 304% with inclusion of Presumed Pathogenic missense variants. In contrast, inclusion of VUS had a comparatively minor impact on the estimated prevalence—a 29–119% increase.

Our estimated genetic prevalence was also 211% higher than a previous study which estimated it to be ~ 1 in 200,000 pregnancies, including all presumed Pathogenic variants [3]. This study had several methodological differences including the use of a smaller population database (the Exome Aggregation Consortium (ExAC)), not using Mastermind to complete a literature review, and no use of ACMG/AMP interpretation [12]. Because of the greater number of individuals represented in gnomAD as compared to ExAC, the number of carriers identified was likely increased based on a more representative statistical sampling for these rare variants. Moreover, a significant number of new variants and patients were reported since the prior publication, resulting in both a greater number of Known Pathogenic/Likely Pathogenic variants (27 compared to 17) as well as an increased number of Presumed Pathogenic variants (170 compared to 96).

Specifically pertaining to the Known Pathogenic/Likely Pathogenic variants, there were 8 variants that were presumed to be Pathogenic in the previous study by assessment of computational predictions but confirmed to be Pathogenic/Likely Pathogenic by ACMG/AMP standards in this study, 3 variants that were classified as Pathogenic/Likely Pathogenic in both studies but were present only in gnomAD, 3 variants that were classified as Pathogenic in the previous study but were VUS by ACMG/AMP in this study, 2 that were presumed to be Benign in the previous study but were found to be Likely Pathogenic in this study, and 1 variant that was not identified in the previous study but was found to be Pathogenic/Likely Pathogenic in this study.

Overall, the assessment of complete literature evidence along with ACMG/AMP interpretation was instrumental in ensuring the precision of the genetic prevalence estimate. If the estimate relied solely on classifications in ClinVar, but otherwise used the same methodology, a total of 15 variants would have been excluded on the basis of conflicting classifications and/or conflicting computational predictions.

**Fig. 2:**
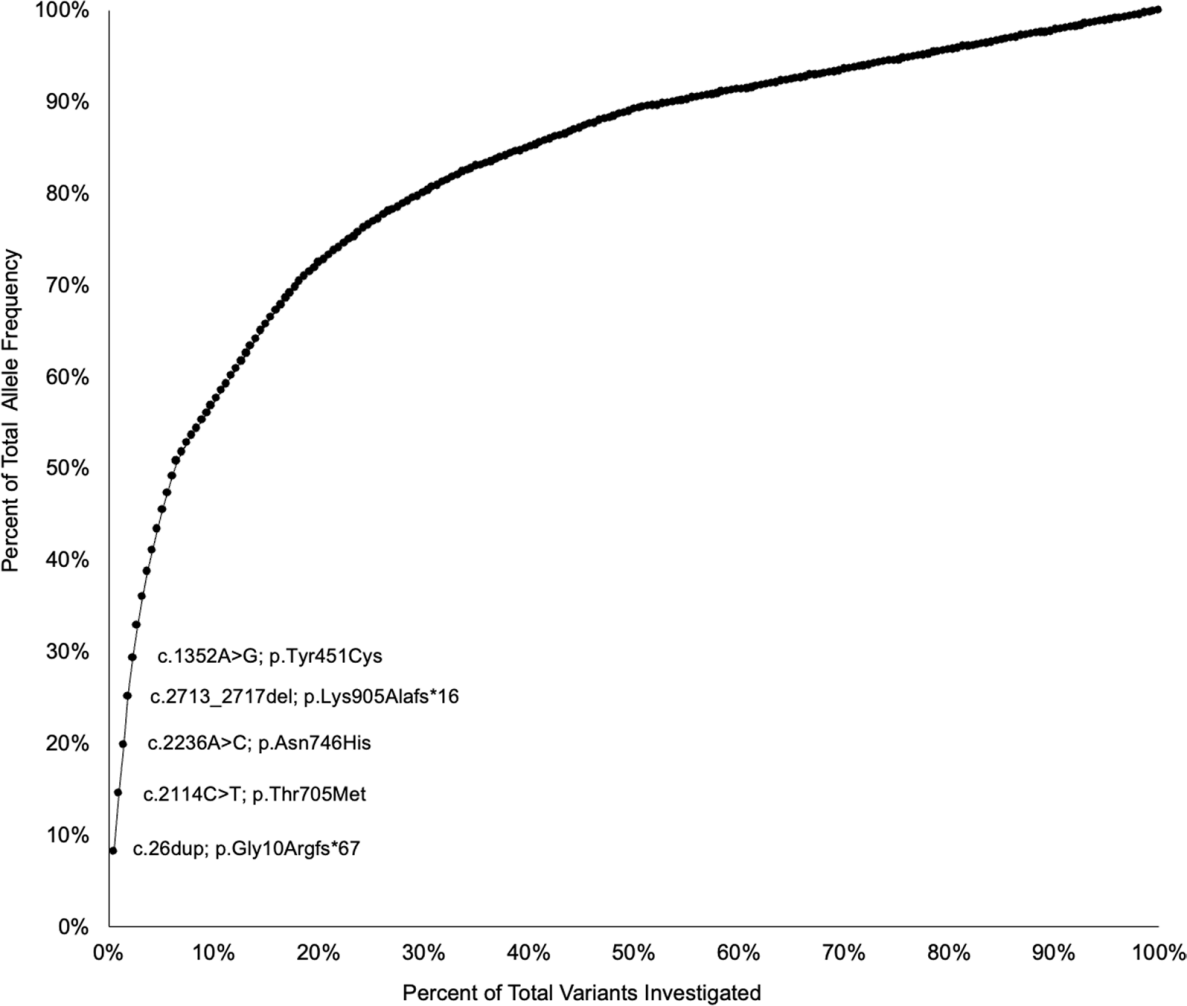
Contribution of Individual *ENPP1* Variants to the Genetic Prevalence Estimate

Assessing the contribution of individual *ENPP1* variants to the genetic prevalence estimate revealed that only 13 variants accounted for 50% of the total allele frequency included in the prevalence calculation (Fig. 2). The top five most frequent variants alone—c.26dup; p.Gly10ArgfsTer67 (0.032% allele frequency), c.2114C > T; p.Thr705Met (0.025% allele frequency), c.2236A > C; p.Asn746His (0.021% allele frequency), c.2713_2717del; p.Lys905AlafsTer16 (0.021% allele frequency), c.1352A > G; p.Tyr451Cys (0.016% allele frequency)—accounted for 30% of the total.

The most common variant (c.26dup; p.Gly10ArgfsTer67) is a previously unpublished variant that is presumed to be pathogenic as a result of it being a frameshift variant at the 5’ end of the gene but may have an inflated allele frequency due to the small number of captured alleles (3,086 overall). The remaining high frequency variants were unaffected by this issue. p.Thr705Met is an unpublished variant that is predicted to be pathogenic by computational algorithms, p.Asn746His has previously been found in four probands initially suspected of having X-Linked Hypophosphatemia (XLH) but is considered a VUS by both our interpretation and interpretations in ClinVar, and p.Lys905AlafsTer16 and p.Tyr451Cys have been found in multiple patients with GACI/ARHR2 and are interpreted as Pathogenic and Likely Pathogenic, respectively [13–16].

**Fig. 3:**
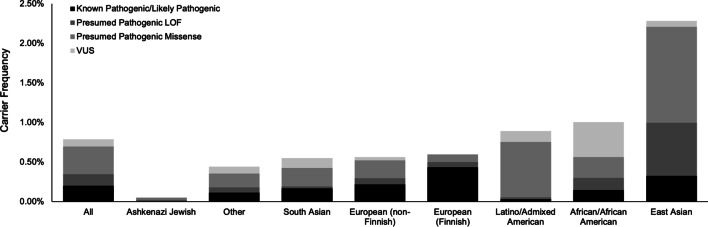
Population-Specific Carrier Frequencies of *ENPP1* Variants. The carrier frequency was calculated using the Hardy–Weinberg equilibrium equation and the sum of the allele frequencies of the specified variants. Known Pathogenic/Likely Pathogenic: variants that were classified as Pathogenic/Likely Pathogenic by ACMG/AMP. Presumed Pathogenic LOF: start loss, nonsense, frameshift, and canonical splice site variants that were found in gnomAD but not the published literature. Presumed Pathogenic Missense: predicted damaging missense variants that were found in gnomAD but not the published literature

In addition, the estimated heterozygous carrier frequency of *ENPP1* variants was found to vary between specific populations in gnomAD, with the East Asian population having a significantly higher carrier frequency than other populations (2.3%; Fig. 3). The most frequent variant in the East Asian population is c.26dup; p.Gly10Argfs*67 (0.32% allele frequency in the East Asian population). As noted previously, this variant was captured in a low number of alleles overall, and 1/310 alleles in this population, which could have artificially inflated the allele frequency, and subsequently the carrier frequency. Removing this variant from consideration, the carrier frequency of *ENPP1* variants in the East Asian population is 1.6%, which remains higher than other populations in gnomAD.

Notably, considering only the Known Pathogenic/Likely Pathogenic variants, the carrier frequency of *ENPP1* variants was highest in the Finnish population at 0.43%, which the next highest being the East Asian population at 0.32% (Fig. 3). The most frequent pathogenic variant in the Finnish population is c.2713_2717del; p.Lys905Alafs*16 (0.18% allele frequency in the Finnish population), which has previously been published in three Caucasian siblings with GACI as well as three unrelated patients with GACI/ARHR2, two of which were American (unspecified ethnicity), and one of which was Finnish [3, 14, 16].

## Discussion

An adequate assessment of the prevalence of rare diseases is crucial for estimating the burden of disease as well as assessing the number of individuals who might benefit from potential new therapies. Rare diseases are often difficult to diagnose which limits the accuracy of epidemiological assessment that is based solely on clinical reports. One recently developed technique that can overcome this limitation involves evaluating the frequency of pathogenic variants in population databases, and then estimating the genetic prevalence of the disease assuming Hardy–Weinberg equilibrium [5–7].

Prior work estimated the genetic prevalence of ENPP1 Deficiency as 1 in 200,000 pregnancies, 3.1-fold less frequent than the current estimate of 1 in 64,000 pregnancies [3]. This discrepancy can be attributed to the identification of additional variants since the prior publication, the use of a 2.3-fold larger population database (gnomAD having 141,456 individuals compared to 60,706 in ExAC), and the use of clinical standard variant interpretation [10, 12]. Thus, our numbers should not be considered definitive, but rather an improved estimate of genetic prevalence based on increased availability of population data and published reports of ENPP1 Deficiency in the five intervening years between these two analyses. Our genetic prevalence estimates could be further improved in coming years, especially with inclusion of data from larger population studies, such as the UK Biobank [17].

This genetic prevalence estimate also has several important caveats that should be considered in terms of its accuracy and impact on diagnosis and therapeutic development for ENPP1 Deficiency. (1) This estimate cannot predict the phenotypic presentation of the disease, given the clinical heterogeneity and a lack of documented genotype–phenotype correlation, and should be interpreted as an estimate of all phenotypes associated with ENPP1 Deficiency; (2) This estimate is only representative of the number of pregnancies with a disease-associated genotype and not a birth prevalence, given the possibility of in utero lethality; (3) A significant number of patients with ENPP1 Deficiency present with novel variants not previously reported in the literature and/or not captured in a population database; (4) A small proportion of ENPP1 Deficiency cases result from structural variants; however, gnomAD lacks complete data on these variants and so they were not considered in our estimate; and (5) Our method of estimating genetic prevalence only takes into account biallelic inheritance (homozygous or compound heterozygous), while recent literature suggests that certain variants can lead to disease even in a heterozygous state [13, 18]. All of these aforementioned factors, as well as additional diagnostic challenges, could lead to a discrepancy between the estimated genetic prevalence and the observed clinical prevalence of ENPP1 Deficiency.

The current genetic prevalence estimate of 1 in 64,000 pregnancies indicates that ENPP1 Deficiency is likely significantly under-diagnosed clinically. In addition, our results indicate that ENPP1 Deficiency may be more common in certain populations, particularly East Asian populations. This estimate, however, is complicated by the relative lack of sequencing coverage in these populations, with only 9,977 genomes or exomes available in gnomAD compared to 64,603 for Non-Finnish European populations. Expanded sequencing efforts in East Asia would be required to confirm this finding.

These results, overall, indicate that expanded genetic testing, especially in non-European populations, will be crucial for ensuring comprehensive diagnosis of patients with ENPP1 Deficiency. The emerging use of Newborn Screening by Next-Generation Sequencing will support this effort by ensuring early diagnosis and mitigating the challenges presented by the clinical heterogeneity and low clinical awareness of the disease.

These efforts to expand sequencing are particularly important now that an enzyme replacement therapy (ERT) is being developed and has been shown to be beneficial in mouse models of ENPP1 Deficiency [4, 13, 18–20]. Specifically, ERT has been shown to be effective in preventing mortality and vascular calcification, improving blood pressure and cardiovascular function, suppressing vascular cell proliferation and intimal hyperplasia, preventing osteomalacia, and partially preventing musculoskeletal comorbidities such as enthesis calcification. Clinical trials of ERT in patients with ENPP1 Deficiency are also currently underway. Accurate and timely diagnosis of patients with ENPP1 Deficiency will be crucial to ensure that patients receive appropriate treatment, should this therapy be approved for clinical use.

## Conclusion

We provided a more accurate genetic prevalence estimate of ENPP1 Deficiency benefiting from a comprehensive literature review using Mastermind, a larger population database, and improved interpretation of variants as per current clinical guidelines. The current estimate of 1 in 64,000 pregnancies suggests that ENPP1 Deficiency is likely significantly underdiagnosed. In addition, the higher carrier frequency observed in East Asian populations illustrates a need for expanded sequencing in non-European populations both to confirm this finding and to ensure maximal and accurate diagnosis.

## Methodology

### Aggregation, classification, and selection of *ENPP1* variants for inclusion in genetic prevalence calculation

Published variants in *ENPP1,* including single-nucleotide variants (SNVs) and indels, along with their corresponding references, were automatically identified and extracted from the medical and scientific literature using Mastermind, a database that is assembled by Genomenon, Inc. [8]. This analysis included systematic review of 1,547 articles describing variants in *ENPP1* published on or before May 23, 2022.

Each variant was standardized using the GRCh37/hg19 genome build and the canonical transcript—*NM_006208.3*—as well as the nomenclature guidelines set by the Human Genome Variation Society (HGVS) [21]. These variants were then manually interpreted according to the standards set by the American College of Medical Genetics and Association of Molecular Pathologists (ACMG/AMP) [9]. This interpretation process considered clinical and functional studies from the literature, population frequencies derived from gnomAD v2.1.1, computational predictions of the effect of missense variants derived from PolyPhen-2, MutationTaster2, and SIFT, and computational predictions of splicing defects for single nucleotide variants derived from dbscSNV [10, 22–25].

Variants identified from the literature were selected for inclusion in the prevalence calculation through consideration of its presence or absence in published ENPP1 Deficiency patients, the classification by ACMG/AMP, the allele frequency in gnomAD, and the classification in ClinVar—a crowdsourced database of variant classifications [11].

Variants that were Benign/Likely Benign by ACMG/AMP and/or were Benign/Likely Benign in ClinVar were excluded. Variants that had conflicting classifications, including those that had sufficient evidence to simultaneously meet both a Benign and Pathogenic classification by ACMG/AMP were also excluded. Remaining variants were included/excluded as specified in Additional file 1: Figs. S1–S2.

Variants that were not found in a patient with ENPP1 Deficiency or were found in gnomAD but not the published literature were also selected for inclusion in the prevalence calculation through consideration of the variant effect type, assessment by Loss-Of-Function Transcript Effect Estimator (LOFTEE), allele frequency in gnomAD, and computational predictions from PolyPhen-2 and SIFT (for missense variants) [10, 22, 25]. The inclusion/exclusion process is specified in Additional file 1: Fig. S3.

### Calculation of genetic prevalence for ENPP1 deficiency

All variants in *ENPP1* were downloaded from gnomAD v2.1.1 along with their overall and population-specific allele frequencies [10]. gnomAD v2.1.1 contains 125,748 exome sequences and 15,708 whole-genome sequences from 141,456 unrelated individuals, which are selected for absence of early-onset disease. Because ENPP1 Deficiency manifests with significant morbidity, we presumed that there are no ENPP1 Deficiency patients represented in the gnomAD dataset.

The overall (or population-specific) allele frequency was summed across all selected variants and then used within the Hardy–Weinberg equation to calculate the carrier frequency (2pq) and the frequency of a disease-causing genotype (q^2^) [26]. This calculation was performed for different sets of variants, segregated by classification and presence in the literature.

## Supplementary Information

**Additional file 1.** Selection Process for ENPP1 Variants in Genetic Prevalence Estimates. Description: Processes for determining whether individual ENPP1 variants will be included or excluded from the genetic prevalence estimates

**Additional file 2. **ENPP1 Variants Analyzed in Genetic Prevalence Estimates. Description: ENPP1 Variants analyzed in the genetic prevalence estimates along with their respective allele frequencies, classifications by Genomenon/ClinVar, and reason for inclusion

## Data Availability

All data analyzed during this study are available in gnomAD (https://gnomad.broadinstitute.org/) and this published article/its supplementary file.
