# Supplementary material for: Estimation of ENPP1 deficiency genetic prevalence using a comprehensive literature review and population databases
Source: Orphanet J Rare Dis. 2022 Dec 2;17:421. doi: 10.1186/s13023-022-02577-2 (PMC9717445; doi:10.1186/s13023-022-02577-2)
Supplement: Supplementary file 1 — Additional file 1. Selection Process for ENPP1 Variants in Genetic Prevalence Estimates. Description: Processes for determining whether individual ENPP1 variants will be included or excluded from the genetic prevalence estimates [file 13023_2022_2577_MOESM1_ESM.pdf]

Supplemental Figures 1-3:
Selection Process for *ENPP1* Variants in Genetic Prevalence Estimates

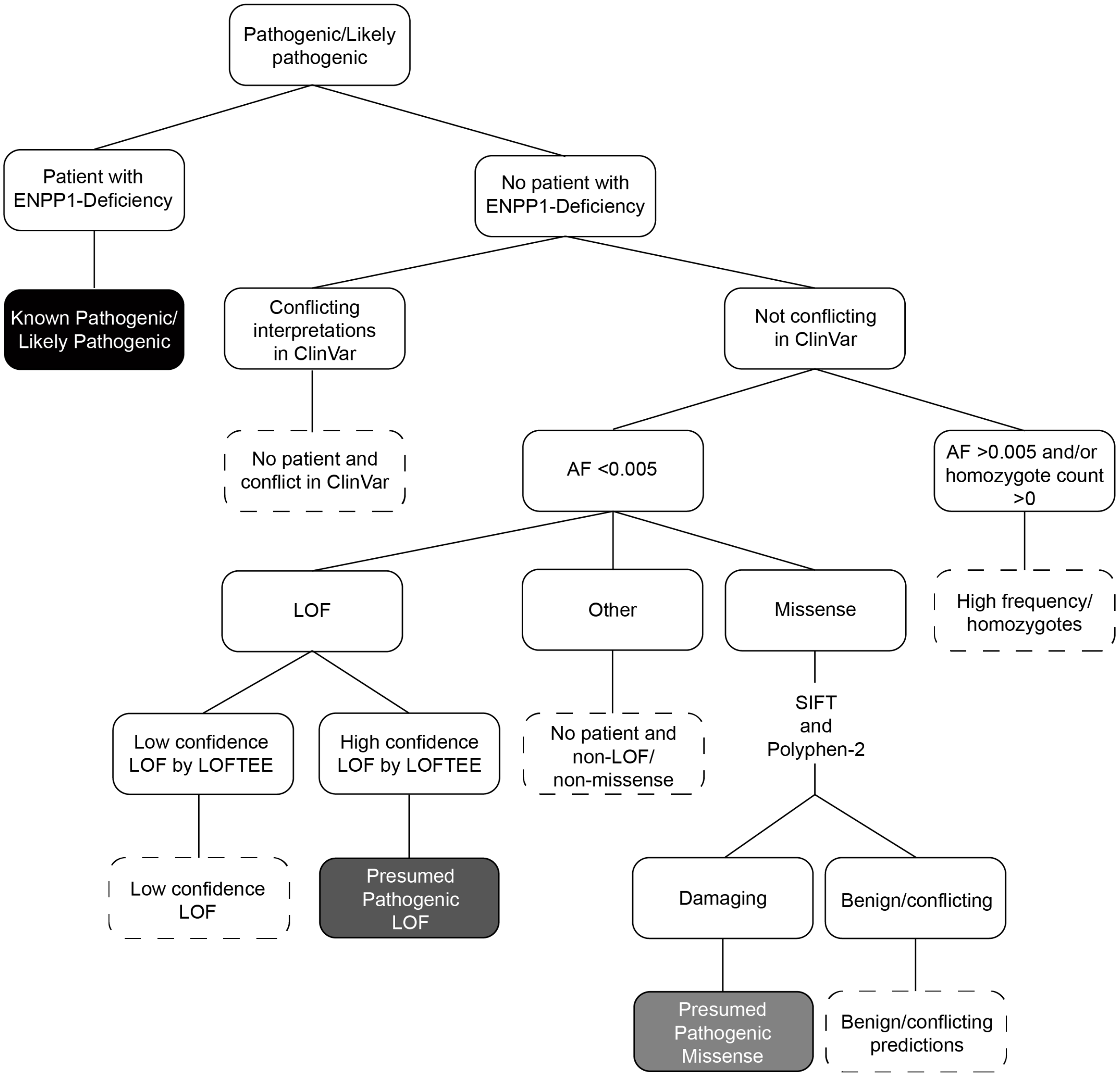

Supplemental Figure 1. Selection Process for Published *ENPP1* Variants Classified as Pathogenic/Likely Pathogenic

Variants must have a non-zero allele frequency in the Genome Aggregation Database (gnomAD) to be included.

Grey/black nodes are variants that will be included in the calculation. Nodes with dashed lines are variants that will be excluded from the calculation. Loss of Function (LOF) variants include nonsense, frameshift, splicing, and start-loss variants.

AF = allele frequency in gnomAD

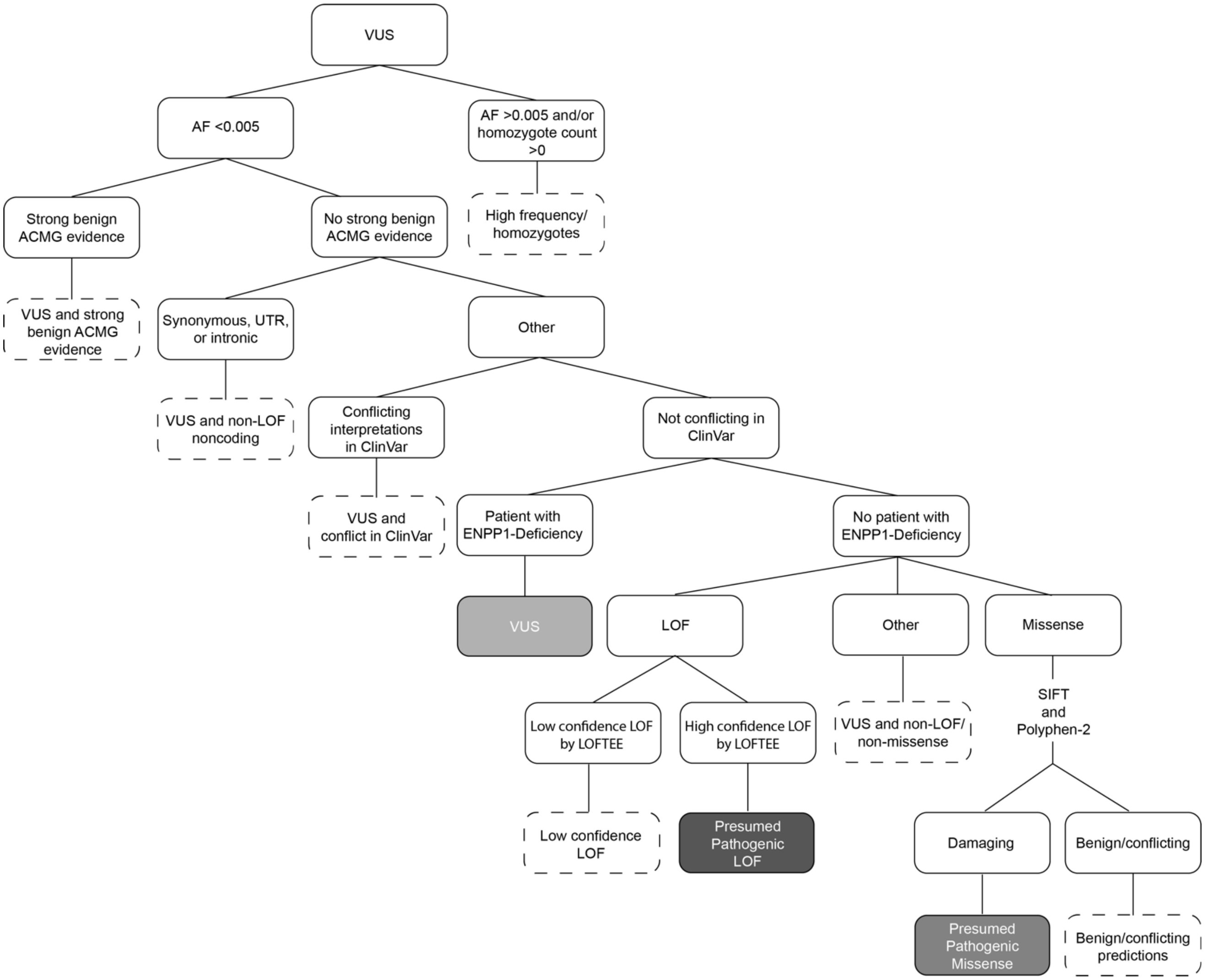

Supplemental Figure 2. Selection Process for Published *ENPP1* Variants Classified as Variants of Undetermined Significance (VUS)

Variants must have a non-zero allele frequency in the Genome Aggregation Database (gnomAD) to be included.

Grey/black nodes are variants that will be included in the calculation. Nodes with dashed lines are variants that will be excluded from the calculation. Loss of Function (LOF) variants include nonsense, frameshift, splicing, and start-loss variants.

AF = allele frequency in gnomAD

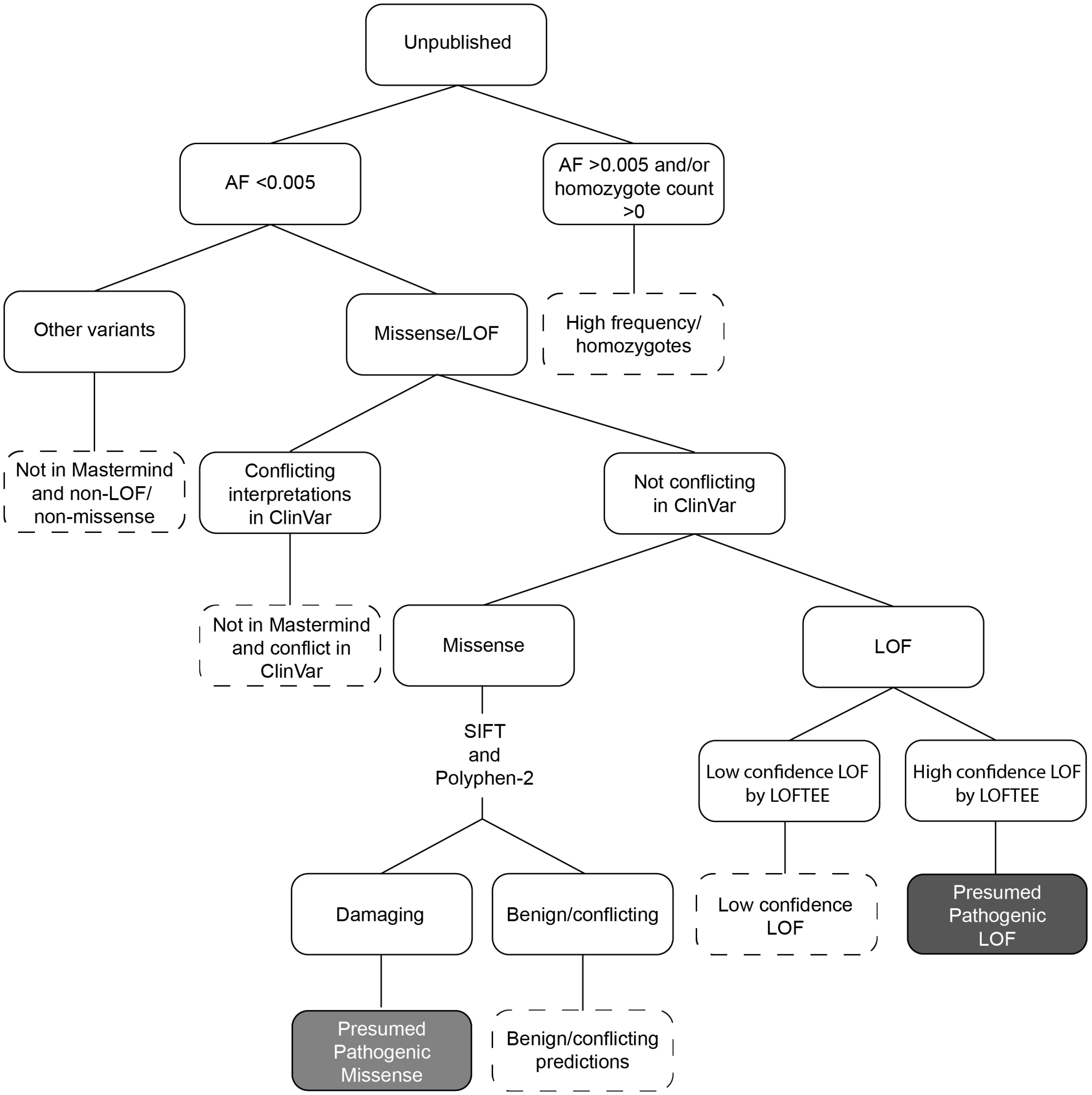

Supplemental Figure 3. Selection Process for Unpublished *ENPP1* Variants

Variants must have a non-zero allele frequency in the Genome Aggregation Database (gnomAD) to be included.

Grey/black nodes are variants that will be included in the calculation. Nodes with dashed lines are variants that will be excluded from the calculation. Loss of Function (LOF) variants include nonsense, frameshift, splicing, and start-loss variants.

AF = allele frequency in gnomAD
